# Transient Attention Gates Access Consciousness: Coupling N2pc and P3 Latencies Using Dynamic Time Warping

**DOI:** 10.1523/JNEUROSCI.1798-23.2024

**Published:** 2024-05-24

**Authors:** Mahan Hosseini, Alon Zivony, Martin Eimer, Brad Wyble, Howard Bowman

**Affiliations:** ^1^The School of Computing, University of Kent, Canterbury CT2 7NZ, United Kingdom; ^2^Cognitive Neuroscience, Institute of Neuroscience and Medicine (INM-3), Forschungszentrum Jülich, Jülich 52428, Germany; ^3^University of Sheffield, Sheffield S1 4DP, United Kingdom; ^4^Birkbeck College, University of London, London WC1E 7HX, United Kingdom; ^5^Psychology Department, Penn State University, University Park, Pennsylvania 16801; ^6^School of Psychology and School of Computer Science, University of Birmingham, Birmingham B15 2TT, United Kingdom

**Keywords:** consciousness, electroencephalography, N2pc, P3, selective attention, working memory

## Abstract

The N2pc and P3 event-related potentials (ERPs), used to index selective attention and access to working memory and conscious awareness, respectively, have been important tools in cognitive sciences. Although it is likely that these two components and the underlying cognitive processes are temporally and functionally linked, such links have not yet been convincingly demonstrated. Adopting a novel methodological approach based on dynamic time warping (DTW), we provide evidence that the N2pc and P3 ERP components are temporally linked. We analyzed data from an experiment where 23 participants (16 women) monitored bilateral rapid serial streams of letters and digits in order to report a target digit indicated by a shape cue, separately for trials with correct responses and trials where a temporally proximal distractor was reported instead (distractor intrusion). DTW analyses revealed that N2pc and P3 latencies were correlated in time, both when the target or a distractor was reported. Notably, this link was weaker on distractor intrusion trials. This N2pc–P3 association is discussed with respect to the relationship between attention and access consciousness. Our results demonstrate that our novel method provides a valuable approach for assessing temporal links between two cognitive processes and their underlying modulating factors. This method allows to establish links and their modulator for any two time-series across all domains of the field (general-purpose MATLAB functions and a Python module are provided alongside this paper).

## Significance Statement

We provide evidence for a temporal link between two important event-related potential components, the N2pc and P3. We establish that the N2pc–P3 link is stronger after correct responses, which provides a new perspective on how links between attention and WM encoding affect the quality of performance and the content of access consciousness. We demonstrate that our dynamic time warping-based method can be adopted to identify yet unknown factors modulating the relationship between two cognitive processes. This method is able to assess temporal links between two time-series of any kind. Thus, it carries the potential to establish a wide range of still unknown temporal links between two cognitive processes (and their modulating factors) across all domains of the field.

## Introduction

The event-related potential (ERP) literature has focused on linking specific cognitive functions with specific evoked components. To gain a fuller understanding of interdependent cognitive functions, it is equally important to uncover associations between ERP components during the performance of particular tasks. This paper seeks to establish such an association, while also providing a general methodological framework (based on dynamic time warping, DTW) to investigate temporal couplings between two time-series (e.g., EEG, MEG, or MVPA). Specifically, we provide new evidence for the functional coupling of two components that have been extensively explored: the N2pc (Eimer, 1996) and the P3 (Polich, 2007). The N2pc is associated with the deployment of attention (Eimer, 1996; Woodman and Luck, 1999) and spatiotemporal selection (Kiss et al., 2008). It is believed to index the transient attentional enhancement (TAE, Li et al., 2017; Zivony et al., 2018) of visual processing triggered by the detection of potentially task-relevant signals. The P3 component has been associated with working memory (WM) encoding and conscious perception (Vogel et al., 1998; Craston et al., 2009; Dehaene, 2014). Despite lingering debate on the origins and function of the P3 (Kok, 2001; Förster et al., 2020; see also Pitts et al., 2014; Shafto and Pitts, 2015), there is widespread consensus that this component reflects high-level cognitive processes that follow attentional selection. In tasks where stimuli are presented in rapid succession (rapid serial visual presentation, RSVP), the P3 is linked to the access of particular stimuli to WM (Bourassa et al., 2015) and conscious awareness (Pincham et al., 2016; Bowman et al., 2022).

Previous studies have obtained initial evidence for temporal links between N2pc and P3 components by demonstrating that experimental manipulations which produce a delayed N2pc often also produce a delayed P3. This pattern was found in attentional blink (Martens and Wyble, 2010; see Zivony and Lamy, 2022 for a review) and distractor intrusion experiments (Botella et al., 2008, Zivony and Eimer, 2021). Demonstrating such temporal links is important, as they might suggest that the cognitive processes associated with the N2pc and P3 (attentional selection and access to WM and awareness, respectively) may also be temporally and functionally linked, in line with models of cascaded hierarchical brain processing (McClelland, 1979). Many computational models (Battye, 2003; Bowman and Wyble, 2007; Olivers and Meeter, 2008; Shih, 2008) have explained temporal links between selective attention and WM encoding or access consciousness with reference to such a cascaded processing architecture.

**Figure 1.**
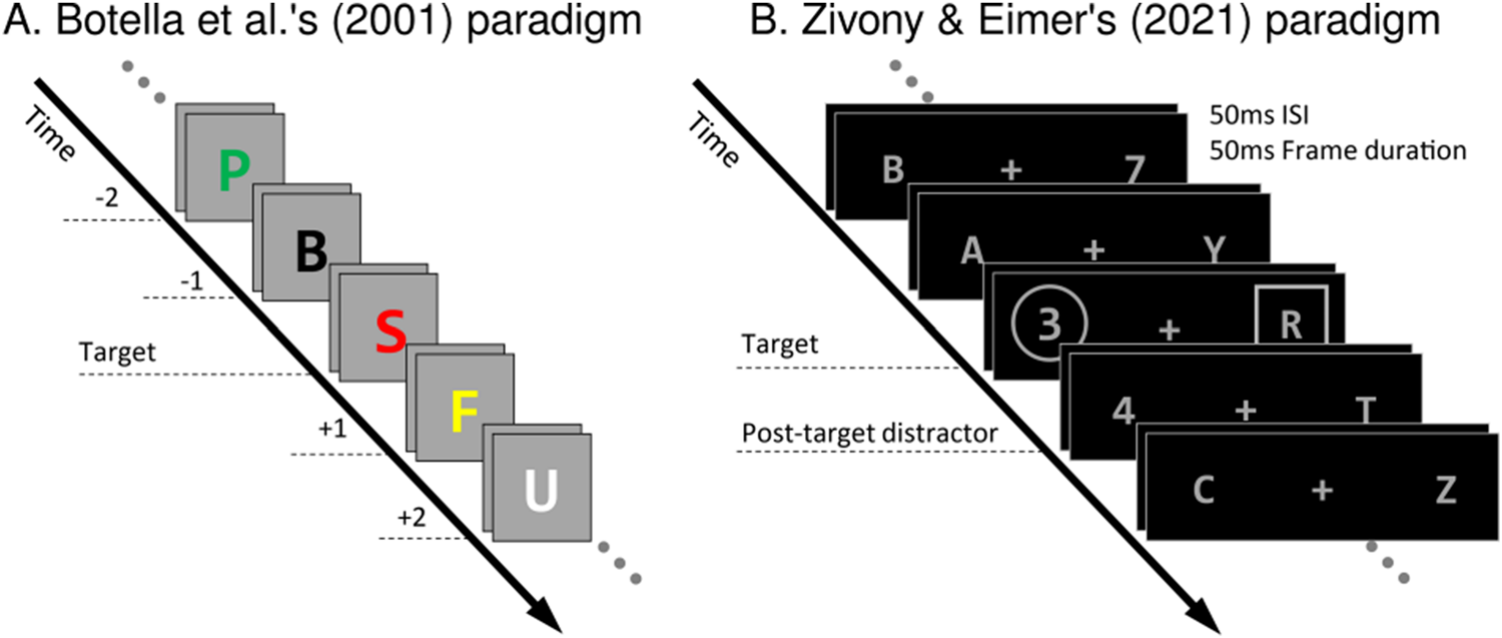
Two example RSVP streams that allow for distractor intrusion errors. Time unfolds from top left to bottom right. In panel ***A***, the task would be to report the red letter. Hence, the illustration depicts the central stimulus set surrounding the target frame that contains the red “S” stimulus, with numbers next to stimulus frames indicating respective item positions with respect to the target (0). Intrusion errors are made if participants erroneously report a neighboring distractor stimulus as being red. For example, a +1 intrusion error is made if the “F,” which immediately follows the red “S” target frame, was reported as being red. Example A is based on the paradigms used by Botella et al. (2001). Example B illustrates the stimulus sequence in Zivony and Eimer’s (2021) Experiment 1. Participants had to report the target digit within one of two RSVP streams, determined by a predefined selection feature (i.e., circle/annulus). The target appeared at positions 5–8 within the stream and was followed by two additional frames. The posttarget frame contained a digit at the same location as the target on 75% of trials and two letters on 25% of trials. ISI, interstimulus interval.

However, most previous studies have measured the latencies of N2pc and P3 components in isolation rather than during the performance of the same task. It has also been shown that these latencies can vary independently, depending on the nature of target selection criteria (Callahan-Flintoft and Wyble, 2017). The goal of this study was to obtain more conclusive, direct, and formally substantiated evidence for temporal associations between N2pc and P3 components. We analyzed ERP data obtained in a previously published RSVP study (Zivony and Eimer, 2021), where observers monitored two lateral RSVP streams to report target digits indicated by a shape cue (Fig. 1*B*). In this task, successful performance required the allocation of attention to the cued object (indexed by the N2pc), followed by its encoding and identification (indexed by the P3). We formally assessed temporal links between these two components, using our DTW framework that can be applied to study associations between any two time-series. Critically, to investigate their functional relevance, we compared these links between trials where the target was reported correctly and trials where a nontarget was reported instead (distractor intrusion). This new approach will enable future research to extend the study of N2pc–P3 links beyond RSVP tasks to other experimental paradigms. It also provides a generally applicable tool (accompanied with the toolbox NeuroWarp, which consists of MATLAB functions and a Python module) to establish temporal links between cognitive processes and their functional roles.

## Materials and Methods

### Experimental paradigm

We will analyze data collected from a lateralized RSVP experiment in the tradition of distractor intrusion experiments (Botella, 1992; Botella et al., 2001; Vul et al., 2009; Zivony and Eimer, 2020, 2021), in which we can identify both N2pc and P3 components. In these studies, participants are asked to detect a single target in an RSVP stream based on a predefined feature (the key feature). Importantly, the target is embedded among distractors that share its reporting feature (the response feature). For example, the target may be the only red letter among differently colored letters (Fig. 1*A*, note that similar paradigms were often used in early experiments; Botella, 1992; Botella et al., 2001). In this case, color will be the key feature as it is used to detect the target, and the identity of the letter is its response feature. In such studies, participants often erroneously report the identity of a distractor that is temporally near to the target and most frequently the immediately following distractor, rather than the target itself (e.g., reporting seeing a red “F” and not a red “S” in Fig. 1*A*).

The present analysis will be performed on the dataset of Zivony and Eimer’s (2021) Experiment 1 (Fig. 1*B*). Zivony and Eimer (2021) conducted an N2pc study (with 23 participants, 16 women; *M*_age_ = 29.43; SD_age _= 9.77) and adopted a dual-stream RSVP paradigm that allowed for intrusion errors of (only) the +1-intruder item (i.e., the distractor item immediately following the target). The main result was that intrusion trials were associated with a delayed N2pc component of lower amplitude.

In their experiments (Fig. 1*B*), participants were presented with two RSVP streams with lengths of 8–11 frames at equal distances from a fixation cross in the center. Gray stimuli were presented in sequence on a black screen, with letters as distractors and digits as targets. The target digit was presented at positions 5–8 of the streams, differentiated by a surrounding annulus or square. Participants had to report the target as accurately as possible after each trial terminated. In target frames, a distractor letter was also presented in the other RSVP stream surrounded by either an annulus or square (which of the shapes identified the target digit was always prespecified). The frame preceding the target frame always consisted of two letters (one in each stream), and earlier pretarget frames were equally likely to contain two letters or one letter and one digit (to ensure that attentional allocation was placed according to the annulus or square rather than alphanumerical category, i.e., participants did not just search for the first digit in the stream). The frame that followed the target frame included another digit at the same location on 75% of trials. In the remaining 25%, a distractor letter was presented instead. Hence, the annulus and the square were the key features in this setting and digit identity the response feature. Each frame was presented for 50 ms, followed by an interstimulus interval (ISI) of 50 ms. Targets were equally likely to be presented in the left or right RSVP stream in each trial.

### EEG data collection and pre-processing

ERPs were computed separately for trials in which participants reported the target digit correctly (correct trials) and for reports of the posttarget digit distractor stimulus (intrusion trials). Incorrect trials, i.e., with reports of neither the target nor the posttarget digit distractor, were excluded in ERP analyses. N2pcs were computed as the contralateral–ipsilateral difference wave between PO7 and PO8 electrodes with respect to the location of the target (e.g., PO8–PO7 if the target was presented in the left RSVP stream). The P3 component was defined as the ERP amplitude at the Pz electrode. Hence, we retained the original paper's (Zivony and Eimer, 2021) EEG methodology in general, with the addition of a 25 Hz low-pass filter for P3s and a larger time window of interest (because the original paper did not analyze P3 components).

### DTW as a measure of latency differences

Our assessment of whether the N2pc and P3 components are temporally correlated uses DTW as a measure of ERP latencies. DTW enables the latency of ERP components to be not based on a given point of the ERP time-series, making it more robust to noise than commonly used point-based latency measures, such as peak latency, fractional peak latency, and fractional area (Handy, 2005; Kiesel et al., 2008; Luck, 2014). For a discussion of the benefits of DTW compared with other EEG latency approaches, see Zoumpoulaki et al. (2015).

DTW measures the similarity between two time-series by aligning/warping one time-series (called the query) to another (the reference). For example, the two time-series in this analysis would be the ERPs from correct and intrusion trials. This alignment is optimal, meaning that a distance matrix is built from all points of the reference and query time-series and a warping path is chosen through this matrix such that the minimal cumulative distance is guaranteed. Grand averages have to be *z* scored prior to DTW to ensure that the warping path just reflects differences in the contours of the reference and query time-series, rather than gross amplitude differences. We further use the area (Fig. 3, shaded in green) between the warping path (Fig. 3, blue line) and the main diagonal (which would indicate identical time series, red line in Fig. 3), henceforth called the DTW area, for our statistical analysis of latency differences between components. Note that standardization via *z* scores as well as using the area for statistical assessment were both proposed by Zoumpoulaki et al. (2015). The DTW area measure indicates succession, as a positive value would imply that the reference time-series (used for alignment, plotted on the *y*-axis in Fig. 3) was overall earlier in time compared with the query time-series (on the *x*-axis in Fig. 3). The DTW area is plotted in light green in Figure 3. We further compute the distance distribution between *x* and *y*-coordinates of the warping path. That is, each (*x*, *y*) coordinate on the warping path has a horizontal distance to the main diagonal. The set of all such horizontal distances gives the distance distribution. The median of this distance distribution allows the computation of components’ latency difference in milliseconds by dividing the median by the sampling rate divided by 1,000 [see Zoumpoulaki et al. (2015) for a formal comparison of the median to other options]. We implemented a time interval of interest of 150–400 ms for the N2pc (Eimer, 1996) and 250–800 ms for the P3 (Polich, 2007). DTW analyses were performed in MATLAB 2020b using the built-in dtw function.

### Placing DTW into statistical inference—permutation test

We assessed statistical significance of these DTW areas with a two-tailed permutation test. We considered a one-tailed test, due to the a priori hypothesis that intrusion trials should lead to later ERP components than correct trials, which was based on the N2pc findings of Zivony and Eimer (2021). However, we decided against a one-tailed test as that would have risked statistical double-dipping (Kriegeskorte et al., 2009), since the dataset upon which the a priori hypothesis was based would be the same dataset as is analyzed by us with DTW. We first implemented the standard paired *t* test permutation procedure, on our participant-level data, where each participant has an ERP for correct and for intrusion. On each iteration of this permutation procedure, a “fair coin” is flipped for each participant; if this comes up heads, the ERPs for this participant are flipped between groups (correct to intrusion and intrusion to correct), if it comes up tails, the ERPs remain as they are. This generates a permuted dataset. We then computed the permutation grand average ERP waves by taking the average wave across participants for (permuted) correct and intrusion conditions separately. We subsequently performed the DTW analysis and computed ERP component DTW areas as described above. We repeated this procedure 10,000 times, which generated a distribution of DTW areas under the null. Finally, the *p* value of our true observed DTW area was computed as the proportion of absolute (hence a two-tailed test) permuted (i.e., null) DTW areas larger than our true observed value. This approach is exactly as proposed previously by Zoumpoulaki et al. (2015), as we used the DTW area value for all statistical analyses and the median of the DTW distance distribution only to estimate components’ latency differences in milliseconds.

### Bootstrap procedure to assess the across-participant variability in the data

We conducted an additional bootstrap procedure to more formally assess our hypothesis of a temporal correlation between human selective attention and WM encoding/conscious perception. This analysis is complementary to the correct versus intrusion comparison which does not reveal a coupling within each condition on its own. The bootstrapping analysis makes this extra inferential step, indicating that within “normal” (i.e., not inducing behavioral change) variability of the electrical brain response, the N2pc and P3s are latency coupled. These analyses were conducted on standardized (i.e., *z* scored) participant-level ERP-components and, identically to our DTW analyses, using a time interval of interest of 150–400 ms for the N2pc and 250–800 ms for the P3. First, we randomly selected participants with replacement 23 times, replicating the number of participants in our other analyses. We then computed bootstrap across-participant grand average ERP waves for our N2pc and P3 components separately. Importantly, the same bootstrap sample of participant replications was used for the N2pc and P3 (that is, if participant *i* appeared *k* times in the N2pc grand average, they also appeared *k* times in the P3 grand average). We subsequently performed a DTW analysis, akin to the one between correct and intrusion trials’ (true observed) ERPs described in the previous paragraph, but now between pairs of true-observed and bootstrapped grand average ERPs. Specifically, we are assessing the latency difference of each bootstrap sampled grand average to the central tendency estimate, which is the true observed grand average. This analysis was conducted separately for the N2pc and the P3 component. It therefore yielded one DTW area measure (relative to the grand average) for the N2pc and one for the P3. We repeated this process 10,000 times and *z* scored the two distributions of DTW areas. Correlation coefficients were then computed after Pearson’s as well as Spearman between the N2pc and P3 DTW area distributions. A significant positive correlation would provide support for our hypothesis of a correlation between the N2pc and P3 components. This is because such a correlation would mean that if the bootstrap N2pc is earlier (or later) than the true observed N2pc, this shift in time translated to the P3 component. To stress, the bootstrap samples were always matched between N2pc and P3 in each of our 10,000 repetitions, pairing N2pc with P3 DTW areas, and enabling the correlations to be calculated. We performed this analysis for correct and intrusion trials separately to prevent possible latency differences driven by the response condition to confound our bootstrap sampling. That is, if intrusion trials should lead to later N2pc and P3 components, some bootstrap samples might show a correlation between the two components just because more intrusion trials were sampled by chance.

### Software accessibility

To increase the value of our methodological approach for the field, we provide the toolbox NeuroWarp, which consists of general-purpose MATLAB scripts and a Python module (the latter using DTW functions of the tslearn toolbox [Tavenard et al., 2020]), alongside this paper. NeuroWarp computes DTW-based latency differences (in milliseconds) as well as temporal correlations between any two time-series. Latency differences can be obtained for between- as well as within-subjects experimental designs. All analyses and figures presented in this paper can furthermore be replicated using an additional set of MATLAB scripts as well as the analyzed ERP dataset. The toolbox, data, and documentation are provided open-source on GitHub (https://github.com/mahan–hosseini/NeuroWarp).

## Results

### Zivony and Eimer’s (2021) Experiments 1A and 1B human ERPs

**Figure 2.**
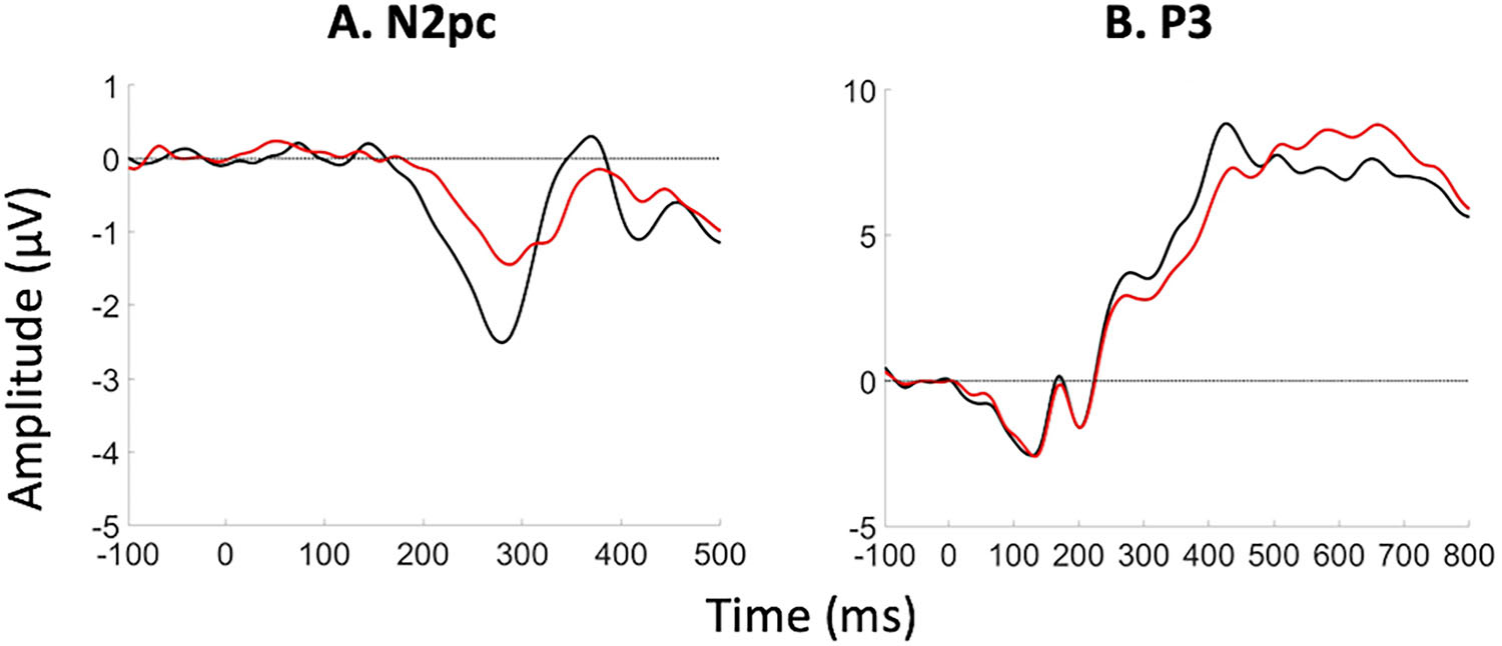
Human ERP data of Zivony and Eimer (2021) Experiment 1. Black and red lines indicate ERPs of correct and intrusion conditions, respectively. We combined the dataset of the authors’ Experiments 1A and 1B, as 1B was a direct replication of 1A.

In Figure 2, we present grand average waves of all 23 participant-level ERPs of Zivony and Eimer’s (2021) Experiments 1A and 1B. In both experiments (1B being a direct replication of 1A), dual RSVP streams were presented, and participants were asked to report the digit target that was surrounded by an annulus. In streams of distractor letters, Zivony and Eimer (2021) only presented either one or two digit stimuli in temporal proximity to the key feature (either the target or the target as well as the immediately following digit [+1 intruder]). Both ERP components, the N2pc (Fig. 2*A*) and the P3 (Fig. 2*B*), qualitatively exhibit latency differences, with intrusion trials showing later ERPs than correct trials. Furthermore, the N2pc (Fig. 2*A*) has a higher amplitude after correct trials (more negative for a negative going effect), which was already noted by Zivony and Eimer (2021). Peak amplitudes of P3 components are comparable but qualitatively occur earlier after correct trials (Fig. 2*B*).

### DTW latency difference analysis of Zivony and Eimer’s (2021) paradigm

#### Replicating the N2pc latency differences

**Figure 3.**
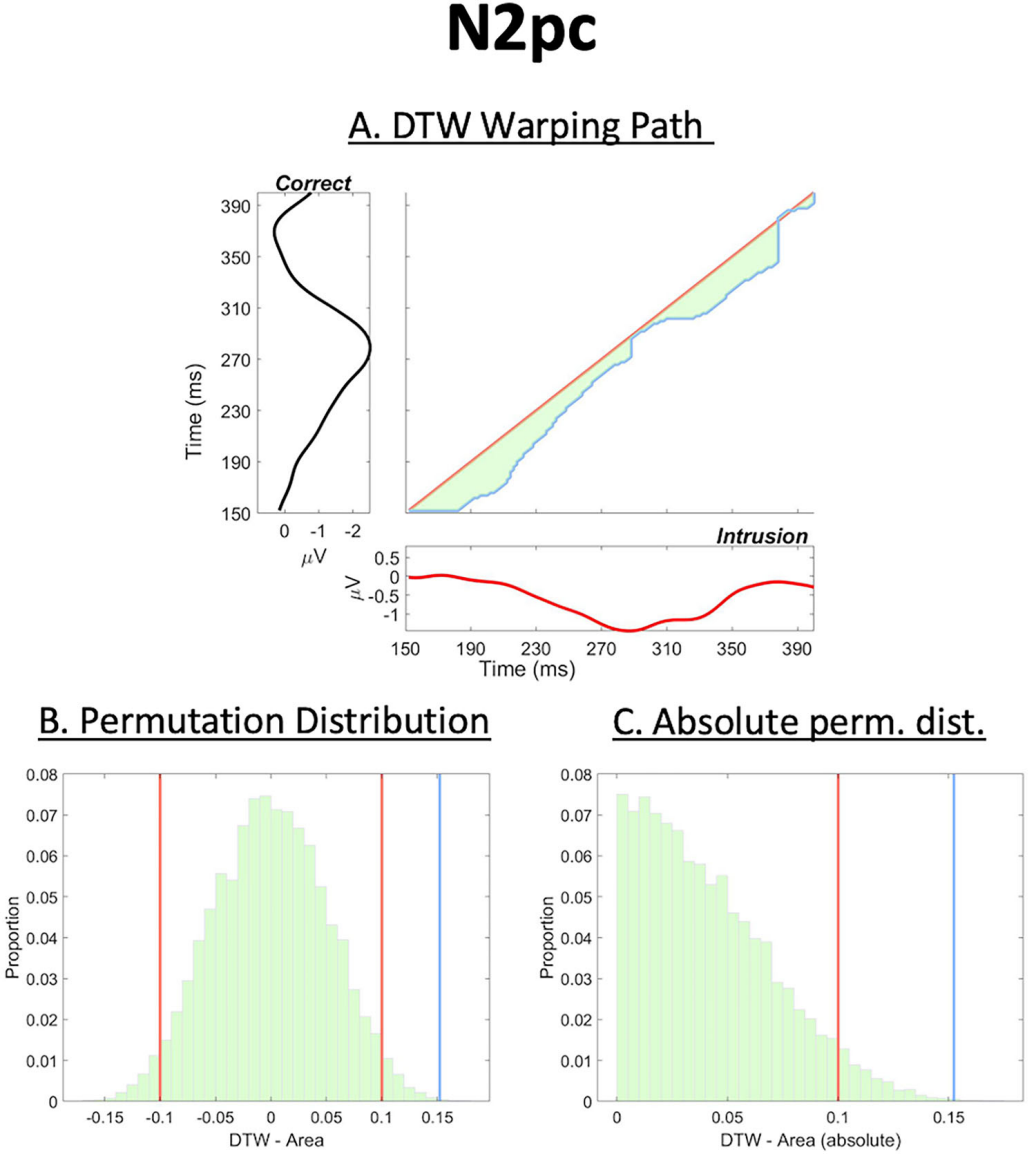
Results of the DTW analysis for the N2pc component. Panel ***A*** presents the warping path (blue), which was found after optimally aligning the reference (correct trials’ N2pc, *y*-axis) and query (intrusion trials’ N2pc, *x*-axis) time-series based on minimal Euclidian distance. The warping path being located under the main diagonal (red) indicates that the reference (correct) preceded the query (intrusion) in time. We found a latency difference of 18 ms using the distribution of distances between all points of the warping path and the main diagonal, which is in line with previous work that used point-based latency estimates. Panels ***B*** and ***C*** display the permutation (null) distribution of DTW areas used for assessment of the latency difference's statistical significance. Panel ***B*** presents the original permutation distribution of DTW areas and panel ***C*** presents these DTW area values after taking the absolute. In order to obtain a two-tailed statistical test, we used the distribution of absolutes presented in panel ***C*** for assessing statistical significance. Red and blue vertical lines in panels ***B*** and ***C*** indicate the threshold of statistical significance and our true observed DTW area value, respectively. We found the latency difference of 18 ms to be significant at an alpha level of 5% (*p *= 0.0013).

**Figure 4.**
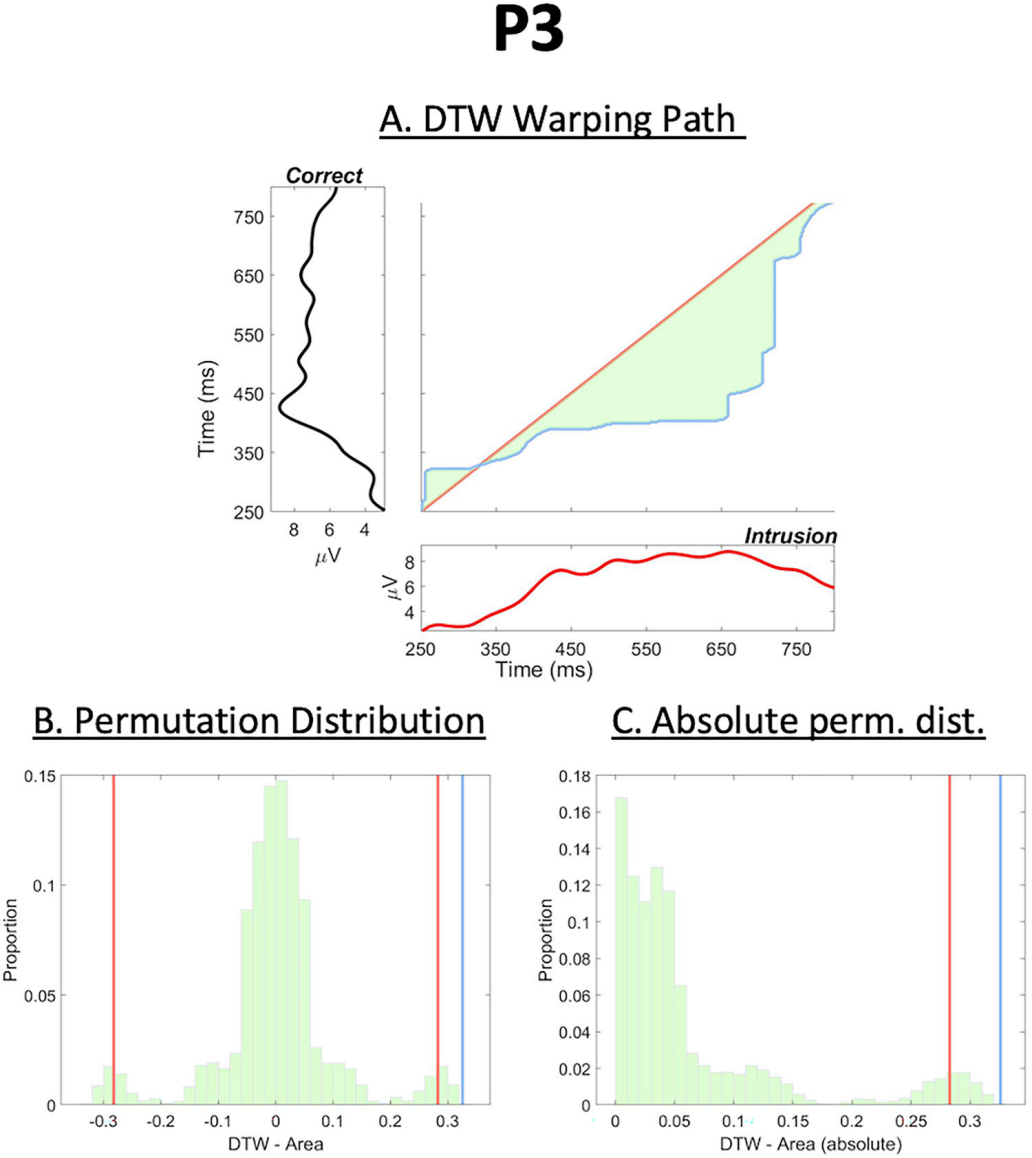
Results of the DTW analysis for the P3 component. Plotting conventions follow those presented in Figure 3, again the DTW warping path as well as the reference and query time-series in panel ***A***, and the original and absolute (null) permutation DTW area distributions in panels ***B*** and ***C***. The P3 component was found to be delayed in intrusion trials by 73 ms, which was again statistically significant (*p *= 0.0003). These results are therefore in line with those presented in Figure 3 and provide initial evidence for the N2pc and P3 component to be correlated in time.

As it is robust against high-frequency noise (Zoumpoulaki et al., 2015), which particularly affects measures of latency focused on individual points, we used DTW to replicate Zivony and Eimer’s (2021) N2pc latency differences between correct and intrusion responses (Fig. 3). We furthermore used the same approach to examine the same latency contrast for the P3 component (measured at Pz, Fig. 4). Figure 3*A* shows the DTW warping path that was found by the algorithm to ensure optimal alignment (i.e., minimal Euclidian distance). We present the DTW reference signal, the N2pc of correct trials, in black on the *y*-axis, and the query signal, the N2pc of intrusion trials, in red on the *x*-axis. We computed the latency difference in milliseconds based on the median of the warping path's distance distribution between *x* and *y*-coordinates, which for the N2pc was 18 ms. This is in line with Zivony and Eimer’s (2021) 50% average peak amplitude criterion, which yielded latency differences of 30 and 20 ms in Experiments 1A and 1B, respectively (note we combined these two experiments into our analysis, as the original Experiment 1B was a direct replication of 1A). It should be noted that the present latency difference of 18 ms is also closely in line with other work by these authors (Zivony and Eimer, 2020), where intrusion trials implied an N2pc component that was 19 ms later than correct trials. The permutation (null) distribution of DTW areas is shown in Figure 3*B*. Our two-tailed permutation test supported our hypothesis that intrusion trials had a later N2pc component than correct trials (*p *= 0.0013). Figure 3*C* shows the absolute DTW area values, which were used to compute this *p* value, as a two-tailed significance test was desired.

#### P3 latency differences at Pz

We further analyzed the latency difference between correct and intrusion trials using DTW for Zivony and Eimer’s (2021) P3 component at the Pz electrode (Fig. 4). We present the DTW warping path and original ERP components in Figure 4*A* and the permutation distributions of latency differences in Figure 4*B*,*C* (we consider the reasons for observing a multimodal permutation distribution later). Again, intrusion trials showed a later P3 component than correct trials, with the latency difference being 73 ms, which was highly statistically significant after running our two-tailed permutation test (*p *= 0.0003).

These findings provide initial evidence for our hypothesis that TAE and encoding into WM are coupled, i.e., a temporal coupling between the N2pc and the P3. This is exactly what we see here: the N2pc and the P3 are both earlier in correct trials. That is, when TAE deployment is earlier (N2pc earlier), encoding is also earlier (P3 earlier). To provide further evidence for this claim, we conducted the following bootstrap analysis.

### Correlation between human N2pc and P3 latencies

**Figure 5.**
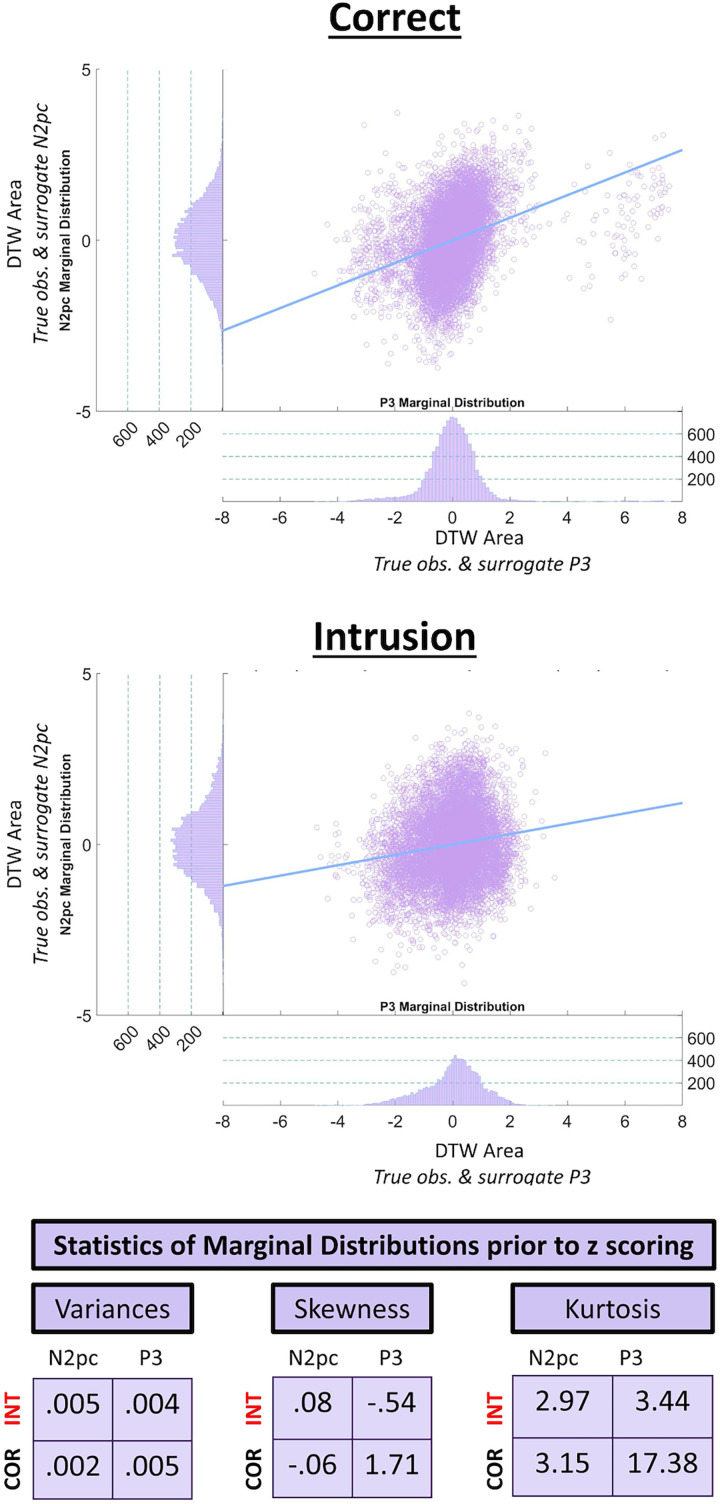
Bootstrap analysis of correlated N2pc and P3 latencies. Top and bottom panels show scatterplots of bootstrapped pairs of *z* scored DTW areas and the line of best linear fit for correct and intrusion trials, respectively. We furthermore present the marginal distributions of true-observed and surrogate ERP-components on their respective axes in both panels. We provide variance, skewness, and kurtosis values of all marginal distributions in the bottom of the figure. In correct trials, the correlation values after Pearson and Spearman were 0.33 and 0.4, respectively. In intrusion trials, the correlation values were 0.15 (Pearson) and 0.14 (Spearman).

In the previous section, we provided evidence that human N2pc and P3 components are affected similarly when moving between behavioral outcomes: correct versus intrusion trials. However, this does not definitively ensure that this coupling obtains when behavioral outcome is constant, i.e., that the coupling obtains due to the intrinsic variability in latencies. This section responds to this aspect by showing that N2pc and P3 latencies are coupled even when behavioral outcome does not change. Figure 5 presents the results of the bootstrap analysis we conducted to probe this hypothesis, which, importantly, was applied to correct and intrusion conditions separately. Figure 5 displays the scatterplots of DTW area pairs with the line of best linear fit as well as two marginal distributions per scatterplot (note we examine the striking outlier cloud on the right of Fig. 5, top panel later). We *z* scored DTW area distributions to obtain a more representational image, i.e., reflecting correlation values more closely, since correlations have internal standardization. We present variance, skewness, and kurtosis values of all four marginal distributions in a table in Figure 5. These values were computed prior to *z* scoring. Both response conditions’ analyses yielded positive correlations between N2pc and P3 DTW latencies after using the same bootstrap samples for the two components in each of the 10,000 bootstrap repetitions. Pearson’s correlations were *r *= 0.33 for correct and *r *= 0.15 for intrusion trials. We provide the rank correlation after Spearman as well to account for the possibility of nonlinear relationships between the DTW area values that were correlated (indeed, we do observe some loss of normality in marginal distributions, see kurtosis and skewness measures below distributions, suggesting heteroskedasticity). Spearman correlations were *r *= 0.4 and *r *= 0.14 for correct and intrusion trials, respectively.

We emphasize that *p* values obtained in resampling analyses of the kind shown here are not really meaningful. This is because the degrees of freedom are determined by the programmer (9,998 in this case) and as discussed in Friston (2012), the fallacy of classical inference states that once the sample size is sufficiently large, *p* values become trivial, as very small effects can become significant. Critically, this does not mean that analyses with many degrees of freedom are inherently flawed but that one should focus on measures of standardized effect sizes, such as correlation coefficients (or differences thereof), when interpreting their results (Lorca-Puls et al., 2018). Stressing that *p* values in the present context do not nearly mean as much as one is used to, all four *p* values associated with the reported correlations were smaller than 0.0001. We also found *p* values smaller than 0.0001 after testing whether the N2pc–P3 correlations were statistically significantly larger in correct trials using Fisher's *Z* transformation. We adopted the equations after Fieller et al. (1957) when testing Spearman correlations.

The positive correlations presented in the previous paragraph support our hypothesis of a temporal correlation between the N2pc and P3 components as well as, more generally, neuroscience's widespread agreement about the cascaded nature of the brain's processing dynamics (McClelland, 1979). Furthermore, our findings support theoretical and computational accounts that postulate a clear link between selective attention and WM encoding/conscious perception. For example, STST models (Bowman and Wyble, 2007) implemented this link architecturally between their blaster circuit (selective attention) and the binding of types to tokens in Stage 2 (WM encoding/conscious perception). Demonstrating such a link empirically in humans is thus important for verifying the conceptual understanding underlying models such as the STST (Bowman and Wyble, 2007). Moreover, we demonstrated a stronger correlation of the N2pc and the P3 components in correct trials, suggesting the presence of factors modulating this temporal correlation, which are considered in the Discussion section later.

### Methodological considerations

Since our present DTW bootstrap procedure constitutes a novel approach to the analysis of neuroscientific time-series data, the following methodological points are important to consider.

#### Do signal-to-noise ratio differences bias DTW?

One methodological concern that might have contributed to the difference in correlations between the N2pc and P3 for correct and intrusion trials focuses on differences in the signal-to-noise ratio (SNR) between the two components. Specifically, compared with correct trials, the decreased amplitude of the N2pc in intrusion trials reflects a lower SNR. The greater influence of noise in participant ERPs will add noise into the DTW. This could lead to an increase in detected temporal variability, over and above any increase in latency variability of the underlying (signal) component. This could, in turn, lead to a reduction in detected correlation of latencies between two components simply because they exhibit increased temporal variability due to reduced SNR.

**Figure 6.**
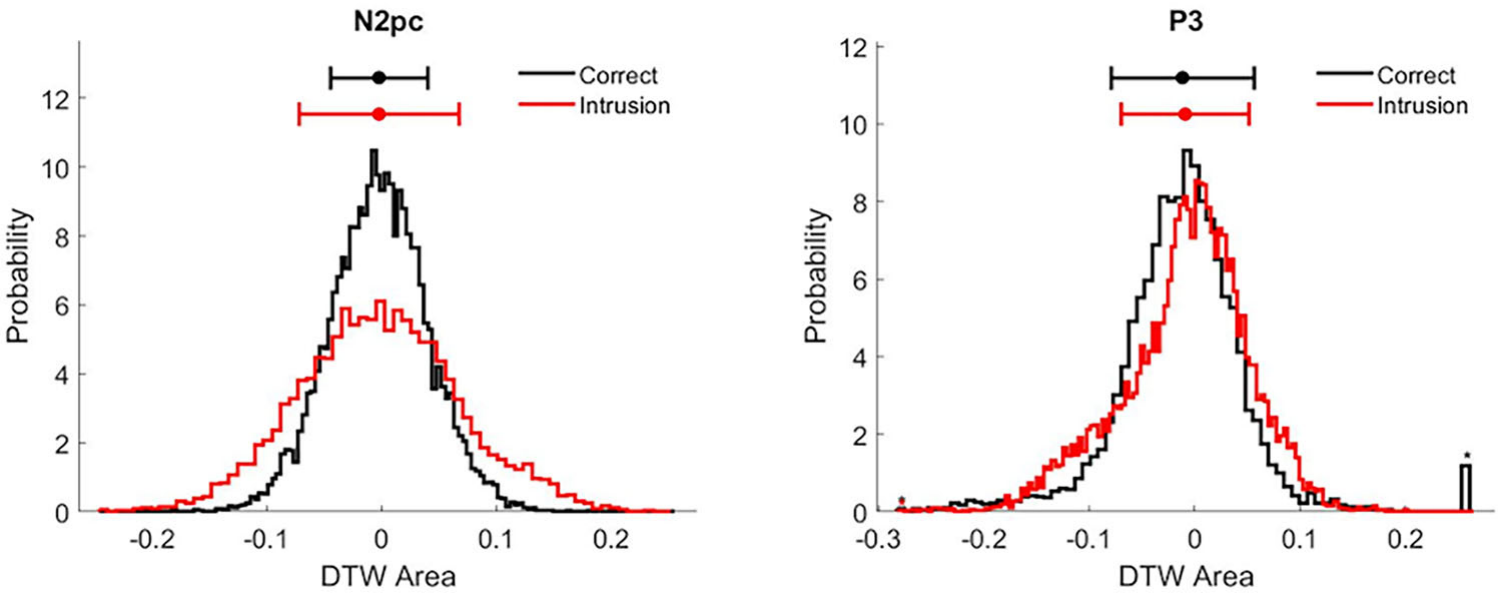
Distributions of *z* scored DTW areas of our bootstrap DTW procedure. Note that these distributions were previously presented as the marginal distribution in Figure 5. As in Figure 5, each value of these distributions measures the latency difference between a given bootstrap GA ERP component and the corresponding true observed GA ERP. Bootstrap samples were kept fixed for analyses of the N2pc and P3. Left and right panels present the marginal distributions of the N2pc and the P3, respectively, and different response conditions are plotted as black (correct reports) and red (intrusion errors) lines. The standard deviations of distributions are plotted as horizontal bars above the corresponding distribution. The dots within these horizontal bars indicate the means, which were all close to zero (due to a given bootstrap ERP being equally likely to unfold earlier or later than the corresponding true observed ERP). The P3 marginals included values ±4 standard deviations, which were not plotted, but are indicated by stars. Particularly large DTW area values in the analysis of the correct P3 are visible as a large black bin on the right. These indicate 86 values (out of 10,000 bootstrap repetitions) that led to DTW area values larger than 0.25 (equaling 4 standard deviations). Note that computing the standard deviations that are presented as horizontal lines above marginal distributions did include these extreme values. Also note that these extreme values were previously evident as a group of outlier points for high *x* values in the scatterplot presented in Figure 5's top panel. We argue that these points furthermore led to the multimodality of the P3's DTW permutation distribution presented in Figure 4*B*.

To investigate this, we present the marginal distributions previously presented on *x*- and *y*-axes of Figure 5, again in Figure 6, now plotting component distributions in separate figures containing both response conditions. These distributions’ variances (i.e., their width; see horizontal bars above distributions for standard deviations) reflect the underlying participant-level ERPs’ temporal variability with respect to the true observed grand average ERP.

The marginal distributions presented in Figure 6, indicate increased temporal variability in intrusion trials for the N2pc, but not the P3, which in fact looks to have reduced variability for intrusions. This finding may be an indication of the SNR decreasing in intrusion trials for the N2pc, which could suggest a reduced capacity to measure the N2pc's latency with DTW in intrusion trials. Such a reduced capacity would add random noise into the measurement of latency, which would have a knock-on effect on the N2pc–P3 correlations, with correlations being weaker in intrusion trials. To investigate this issue, we conducted the following simulation analysis in which a known latency shift was added to the grand average N2pc of intrusion trials. Different levels of noise were then added to this shifted N2pc time-series (to modulate SNRs) and the capacity of DTW to uncover the known latency shift was assessed.

**Figure 7.**
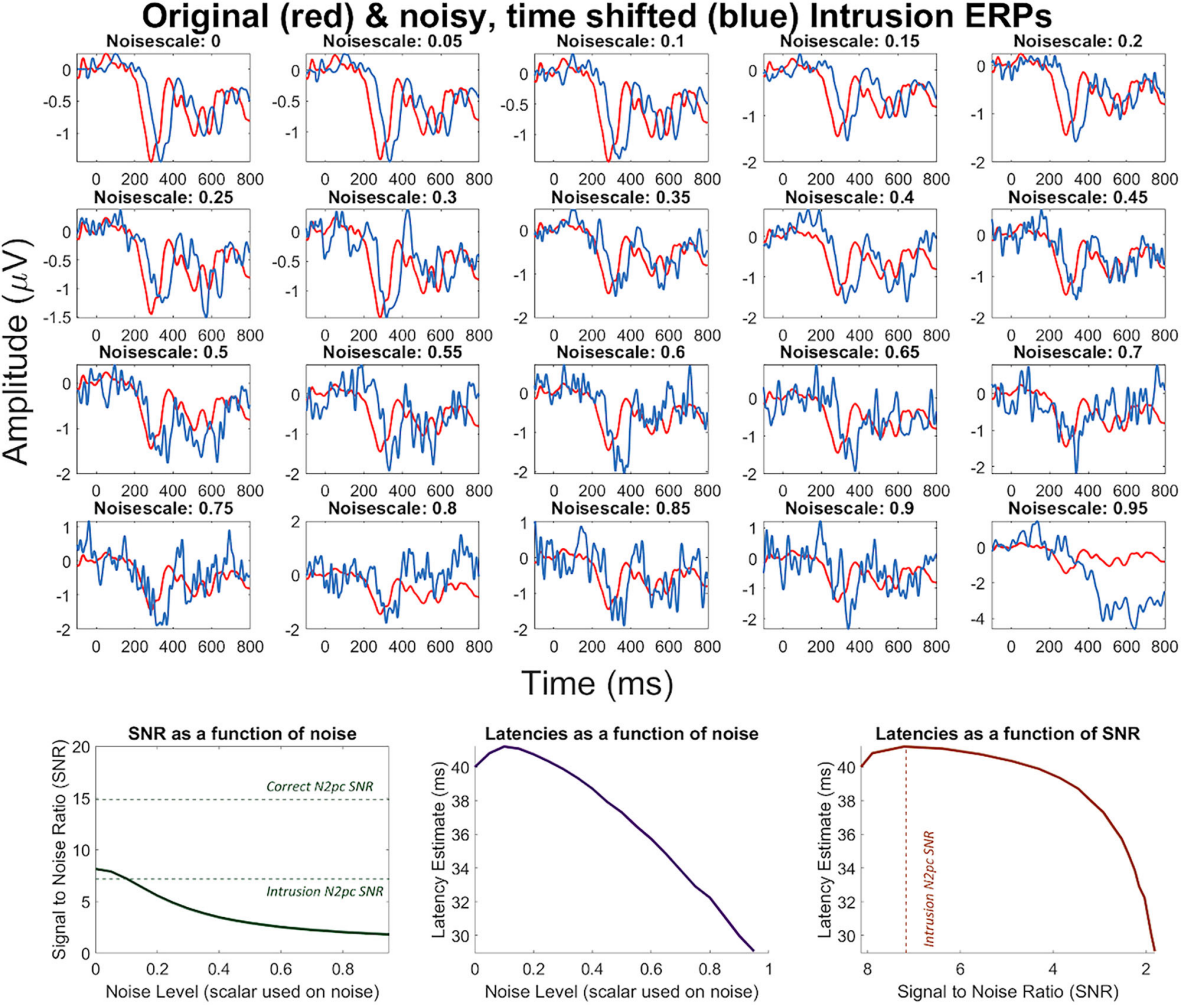
DTW simulations with noise scalars ranging from 0 (i.e., no noise, only the latency shift) to 0.95. The top panels plot the original intrusion N2pc in red and the shifted N2pc at each noise level in blue. Note, the changes in *y*-axis scales as noise amplitude increases. The bottom left panel plots signal-to-noise ratios (SNRs) as a function of the noise level, with the SNRs of correct and intrusion N2pcs plotted as dashed lines. The bottom middle panel plots the latency estimate after DTW as a function of the noise level and the bottom right panel plots the latency estimate as a function of SNR, plotting the intrusion N2pc's SNR as a dashed line. The robustness of DTW to noise levels associated with the intrusion SNR suggest that the differently strong N2pc–P3 correlations after correct and intrusion trials were likely driven by differences in the cortical processes generating an intrusion or correct response.

We first added a shift of 50 ms to the grand average N2pc in intrusion trials, i.e., the latency shifted time-series (henceforth called shifted N2pc) unfolded with a delay of 50 ms. A random noise time-series, based on the human EEG frequency spectrum according to Yeung et al. (2004), was generated. This noise time-series was multiplied by a scalar that ranged from 0 (i.e., just the latency shift and no noise) to 0.95. Figure 7's top panels depict the intrusion N2pc in red [which in all analyses (and, thus, plots) was the original grand average N2pc in intrusion trials] and shifted N2pcs (blue) in the noise scalar range of 0–0.95. After extracting the time window of interest (150–400 ms), we standardized (i.e., *z* scored) intrusion and shifted N2pcs, computed DTW between them and stored the latency estimate as well as SNRs. SNRs were computed as the root mean squared value between 200 and 400 ms divided by the root mean squared value between −50 and 100 ms. For each noise level, this procedure was repeated 25,000 times and average latency estimates as well as shifted N2pcs’ average SNRs were computed.

Figure 7's left bottom panel plots average SNRs as a function of noise level. As noise levels increased, SNRs decreased from 8.14 to 1.8. Note that the SNRs of correct and intrusion trials’ grand average N2pcs were 14.86 and 7.17, respectively, and are plotted as dashed green lines. Figure 7's middle bottom panel plots the latency estimate in millisecond as a function of noise level. It can be seen that without noise (noise level, 0), DTW underestimates the added latency shift of 50 ms by 10 ms while only underestimating it by 8.78 ms for a noise level of 0.1. We understand this outlier to be due to the fact that if no noise is added, the first 50 ms of the shifted N2pc are all zero. This affects the *z* scoring, which in turn affects the DTW estimate to be lower. As the noise included in the shifted N2pc increases, DTW underestimates the latency difference of the two time-series progressively more, being 29.1 ms for the noise level of 0.95. Finally, Figure 7's bottom right panel plots latency estimates as a function of SNRs. The dashed line in this plot now only indicates the SNR of intrusion trials’ N2pcs, since that of correct trials was too large to be included. It can be seen that (with the exception of the low noise outlier points already present in Fig. 7, left panel) as SNRs decrease, DTW underestimates the latency differences between the two time-series progressively. This plot indicates that for SNRs above 4, the latency estimate was underestimated by 10 ms.

This was critical to see, since the SNR of the intrusion N2pc, which was the time-series of our main analysis that was suggested to suffer from increased temporal variability, was in this SNR range with a value of 7.17. Critically, the main issue to assess with these simulations is the difference in efficacy of DTW to measure latencies given that one time-series has an SNR of 14.86 (correct N2pc) and the other of 7.17 (intrusion N2pc). While the simulation results presented in Figure 7 indicate that a difference in estimating latencies with DTW indeed does exist as SNRs decrease, we would argue that this difference is negligible for the main analysis of this paper.

#### Large P3 DTW areas

The scatterplot of (bootstrapped) DTW area values for correct trials presented in Figure 5's top panel shows a group of outlier points for high *x* values. These points are observable as a low amplitude mode high in the *x*-axis marginal distribution, suggesting that this distributional discontinuity is driven by a step change in the DTW values measured for P3s when participants respond correctly. Pursuing this pattern, it became apparent that there are specific (atypical) participant ERPs that will sometimes dominate in a bootstrap sample leading to the bootstrap and true observed P3s showing much larger DTW area values. This is because the DTW warping path has to be considerably further from the main diagonal to align the waveforms. This reveals a step change in the pattern of P3 brain responses. Indeed, this step change in warping paths is surely the phenomenon that underlies the multimodal permutation distributions observed in Figure 4. This multimodality is (as for the bootstrapping) observed for the P3 component. Since the permutation procedure is swapping between conditions, this multimodality could be fully driven by a phenomenon in the correct condition. That is, the negative lobe in Figure 4*B* could be generated when the atypical (correct condition) P3 trials are prominent in one condition (surrogate intrusion) and the positive lobe when those same trials are prominent in the other condition (surrogate correct). Note that this same phenomenon is also observable in Figure 6's right panel as a high amplitude bin for high *x* values.

We reran our main analysis (Fig. 5) after excluding DTW areas as outliers if they exceeded ±5 standard deviations from the mean. Doing so only excluded the low amplitude mode high in the *x*-axis marginal distribution of Figure 5's top panel. A total of 76/10,000 values were excluded. We ensured that if a given DTW area value was excluded from the P3 marginal distribution, the corresponding N2pc DTW area value (i.e., the value that was generated in the same bootstrap repetition) was removed. Correlation values for the analysis of correct trials were *r *= 0.34 and *r *= 0.38 after Pearson and Spearman, respectively. Since no ±5 standard deviation outliers were present for the analysis of intrusions, intrusion correlations are not stated again. This suggests that the correlations presented for correct trials in the main analysis were not driven by the outlier points for high P3 DTW area values.

We do not view this feature as problematic (especially since we provide Spearman's correlation, which is robust to heteroskedasticity and our resampling procedures do not require a normality assumption). Instead, such a pattern could be of theoretical interest. If, for example, those participants that led to a large DTW area value for the P3 when bootstrap sampled often would demonstrate some interesting type of behavior or feature in their P3s, further (theoretically informative) observations could be obtained. This issue therefore reveals a strength of our bootstrap DTW procedure, since such further observations about individual differences would not have been detected otherwise (i.e., with analyses conducted only on grand average ERP latencies).

#### Analyzing the participant-level

Finally, we have focused on across-participant latency variability, rather than across-trial variability. This is because it is difficult to accurately measure component latencies at the single trial, or even the individual participant, levels, because of low SNR. Bootstrapping participants enables us to measure latencies at the grand average level, i.e., bootstrapped samples of participants are assessing the variability around the grand average, with all samples built from ERPs, indeed, as many ERPs (although, of course, with some repeated and some missing) as there are participants. This focus on across-participant (rather than across-trial) variability leaves the possibility that the coupling of N2pc and P3 latencies might arise simply because there is variability in the processing efficiency of different participants’ visual systems. That is, the N2pc and P3 might both be delayed for a participant simply because that individual possesses an inefficient visual processing pathway. However, if such a phenomenon was present, it should also generate a substantial N2pc–P3 latency correlation for errors. That is, the fact that this correlation is substantially higher for corrects than for errors suggests that there is a coupling of N2pc and P3, which is “over and above” any correlation of latencies that might be present due to individual differences in efficiency of visual processing pathways.

Nonetheless, we cannot make claims about the N2pc's and the P3's relationship on the trial level. For a participant with an early N2pc and P3, it could have, for example, been the case that on some trials the N2pc occurs fast, while on other trials, the P3 occurs fast. Aggregating trials, one might then conclude that for this participant the P3 occurs fast when the N2pc does. Although, while we would contend that such a conclusion is the most likely strictly, we cannot make it based upon the analyses performed in this paper. It is important then that our results are extended with a measure that has a better SNR than EEG allowing analyses at the trial level.

## Discussion

This study provides the first formal evidence for a temporal association between the N2pc and P3 components. This evidence is based on the ERP data of a distractor intrusion experiment (Zivony and Eimer, 2021) in which performance required allocation of attention to the cued object (N2pc), followed by its encoding and identification (P3). Using DTW, we initially demonstrated that compared with correct reports, both the N2pc (18 ms, Fig. 3) and the P3 (73 ms, Fig. 4) components occurred later with intrusion errors. Using a participant-level bootstrap DTW procedure, we then provided evidence that the two ERP components are correlated in time within each behavioral outcome (i.e., correct or intrusion trials, Fig. 5). This bootstrap DTW analysis demonstrates the utility of our new method for studying temporal correlations between two time-series. Importantly though, due to the correlational nature of this analysis, statements about causality are more difficult to justify.

### Attention and access consciousness

There is a long debate on the relationship between attention and access consciousness, with, for example, Lamme (2003) arguing that they are independent processes. Our findings may contribute to this debate, if one can make a clear association between access consciousness and the P3. We argue that such a connection can be made in the limited context of RSVP experiments.

RSVP streams bombard the visual system with stimuli, some of which break through into consciousness. Importantly, in such breakthrough experiments, a target-evoked P3 is largely absent when participants cannot report the identity of a target (Sergent et al., 2005; Craston et al., 2009). If participants report the identity of a following distractor, a distractor-evoked P3 emerges instead (Bourassa et al., 2015). These findings suggest that the P3 in RSVP experiments is closely associated with WM encoding. Experiential blink studies (Pincham et al., 2016; Bowman et al., 2022) provide further support for an association between P3 and access consciousness. Specifically, Pincham et al. (2016) provided evidence that in RSVP, P3 amplitude varies considerably more with percept strength (i.e., conscious perception) than with report accuracy.

Alternative interpretations of the P3b, such as Pitts and colleagues’ (Pitts et al., 2014; Shafto and Pitts, 2015; Sandberg et al., 2016) postperceptual account, are typically motivated from non-RSVP experiments. There are important differences between our and Pitts et al. experiments [see Pincham et al. (2016) for a similar discussion]. Most notably, Pitts et al. used no masks in their experiments and therefore their targets were not likely to be rapidly overridden by competing stimuli unless they were immediately encoded. It is likely that interpretation of the P3 is task dependent. While various accounts of the P3 remain possible in various visual search tasks, we contend that the P3 is tightly linked with access consciousness and can be used as a marker of this process in the specific context of RSVP experiments.

On this basis, we suggest that our analyses directly couple attention and access consciousness, suggesting that they are tightly intertwined and far from (statistically) independent. Importantly, the suggested temporal link between the N2pc and P3 supports theoretical and computational models that emphasize a functional relevance of selective attention for WM encoding/conscious perception; e.g., the theories of Zivony and Eimer (2020, 2021) as well as STST computational models (Bowman and Wyble, 2007; Wyble et al., 2009; Chennu et al., 2011; Bowman et al., 2022) and other attentional gating models (Battye, 2003; Olivers and Meeter, 2008; Shih, 2008).

### A need for caution

Our findings are, of course, statistical in nature. Consequently, there is no absolute certainty that attentional selection (N2pc) always precedes access consciousness (P3). Thus, a claim that attention is, in an absolute sense, necessary and sufficient for conscious perception is beyond the scope of our findings. Further, our findings are focused on a specific experimental paradigm. Additional research is needed to investigate the N2pc–P3 link in other experimental designs.

Consistent with the conventions of the field, we asserted that a given ERP component indexes the timing of a certain cognitive process. However, the N2pc should not be taken as indexing the exact onset and offset latencies of attentional enhancement. This is due to the indirect relationship between cortical activity and the signal recorded at EEG electrodes, which measure a dynamic and convoluted wave of activity spreading across tissue and because all cognitive and neural processes unfold gradually in real time.

Notably, a number of theories postulate that the N2pc “drives” the P3 (e.g., the STST theory; Bowman and Wyble, 2007). If such an N2pc–P3 relationship were true, one might wonder why a temporal delay is often observed between P3 and N2pc [even though in the current data the N2pc (∼200–400 ms) overlaps at least partially with the P3; Fig. 2]. One reason could be that the N2pc's activation has to build up before it can drive the P3. Indeed, one interpretation of evoked responses is that they reflect current (the time derivative of membrane potential), rather than membrane potential/activation itself (Murakami and Okada, 2006). Relatedly, another computational model of the N2pc posits that it marks the initiation of attention locking on to the target, and therefore the effect of attention on higher level processing would begin only after the end of the N2pc (Wyble et al., 2020). The observation that the P3 positivity overlaps with the negative rebound of the N2pc (Fig. 2) is then interesting, since the time-derivative interpretation of ERPs suggests that the N2pc neurons would still be active when its deflection has gone negative, it is just that the neurons’ activations/membrane potentials would be decreasing.

### The N2pc–P3 link's function

Our finding of a stronger link between the N2pc and the P3 after correct reports fits previous literature and hints at a possible functional role of the link. In the context of the two-feature STST (2f-STST) model, in which the detection of the target key feature drives attentional enhancement and is indexed by the N2pc, Chennu et al. (2011) argued that the strength of the target's key feature representation plays a central role in resolving response feature competition. If the target key feature is strong, processing would occur quickly and with high amplitude, increasing the likelihood of correct reports and a vivid percept. In contrast, a weak target key feature would lead to increased ambiguity and uncertainty, resulting, more often, in intrusion errors. The P3 indexes the resolution of the 2f-STST's response feature competition and consciously perceiving the winning stimulus. According to this framework, there is an optimal timing between attention and access consciousness that depends on the timing of the target key feature driving TAE: if this occurs when the correct response feature is strong, one observes a larger correlation between the N2pc and P3 and an increased likelihood of a correct response (Fig. 5, top panel). In contrast, if TAE is deployed when the correct response feature is weak, one obtains a closer (i.e., more contested) response feature competition. This, in turn, is more likely to lead to intrusion errors and increased temporal intertrial variability, leading to a lower N2pc–P3 correlation (Fig. 5, bottom panel).

This argument is supported by participants reporting lower confidence after intrusion errors (Recht et al., 2019; Zivony and Eimer, 2020). This is likely to be the result of a (relative to correct percepts of target stimuli) more ambiguous percept. There is, though, the possibility of a third area that is earlier in the processing pathway, and which drives the N2pc as well as the P3, but without any meaningful link between the two. However, a number of points stand against this possibility: (1) we are not aware of a component observed in RSVP that is earlier than the N2pc and varies with behavior, although components can be present to which EEG is blind. (2) It would seem nonadaptive if two such prominent brain responses were not part of a cascade; all major theories of the brain assume cascaded processing along the ventral processing pathway. Additionally, the association of the N2pc with attentional deployment is pretty well accepted, as is the position that the P3 is associated with higher cognitive processing, e.g., conscious perception, WM encoding, or response preparation. It is difficult to see how any of these processes would not be driven by attention.

### Identifying factors modulating the brain's cascaded processing with DTW

The present analysis has the potential to identify factors that contribute to the extent of temporal correlation between cognitive processes (additionally, because DTW can accommodate compressions and expansions in time, these temporal associations can be different to those observed with traditional functional connectivity), enabling novel insights into the cascaded nature of the brain. Additionally, the method could be applied to time-series resulting from other research contexts and using non-EEG measures, e.g., from machine learning or fMRI. For example, a clinician might demonstrate that in a specific patient population, a temporal link between two cognitive processes is weakened and associated with symptom severity. It could then be investigated whether some treatment known to improve symptoms achieves this by modulating the temporal link established with our DTW procedure.

## Conclusion

We have provided novel insight into the nature of links between attention and higher-order cognition, thereby providing evidence against these two processes being independent from or identical to one another. This link was studied using DTW embedded in a bootstrap procedure, which can in general be used to study the temporal link between two components obtained with neuroscientific measures. Applying this approach to the N2pc and P3 ERP components recorded in an intrusion error experiment, we not only provided evidence of a link between selective attention and access consciousness but also suggested that the timing and precision of attentional selectivity likely affects the timing and contents of conscious perception. We furthermore demonstrated that this link has differential strength when correct reports, compared with when intrusion errors, are made, suggesting that the relationship between the N2pc and the P3 is functionally relevant. Our stronger N2pc–P3 link in correct trials complements the literature on distractor intrusion errors, introducing the possibility that the likelihood of “good fortune” (i.e., the correct two features happening to be coactive and encoded together, resulting in a correct response) might be indexed by how tightly selective attention and access consciousness are linked. Still, further research is needed to study the N2pc–P3 link in additional experimental settings to provide a more comprehensive understanding of the two components and their relationship with each other. Future research should also test whether this link has a similar (or different) functional relevance for different cognitive, sensory, and clinical phenomena (e.g., considering different modalities, multimodal integration, motor processes, or impaired processing after neurological disorders).
